# Holding but not folding: How a single charge flip uncouples the DNAJC7‐Hsp70 relay in amyotrophic lateral sclerosis

**DOI:** 10.1111/febs.70472

**Published:** 2026-02-23

**Authors:** Tsung‐Sheng Chiang, Jerome Boisbouvier, Lauren A. Gandy

**Affiliations:** ^1^ University of Grenoble Alpes, CNRS, CEA, Institut de Biologie Structurale (IBS) France

**Keywords:** ALS, chaperone, foldase, holdase, methyl‐NMR, mutation

## Abstract

Genetic mutations impact protein function through various routes: Some catalyze new oncogenic activities, while others trigger complete structural collapse. However, the E425K mutation in DNAJC7, associated with Amyotrophic Lateral Sclerosis (ALS), presents a far more subtle and intriguing case. In their recent study in The FEBS Journal, Elmaleh *et al.* (2026) *FEBS Lett* employed high‐resolution NMR to demonstrate that this mutation leaves the protein's overall structure intact while selectively paralyzing its ability to communicate with the Hsp70 chaperone machinery. In this commentary, we show how their work complements *in vivo* studies that investigate ALS disease pathology at pathway complexity and defines a new target to rescue non‐functioning Hsp70 chaperone systems.

## Graphical abstract

In this issue, Elmaleh *et al.* demonstrate that the E425K mutation in DNAJC7, associated with amyotrophic lateral sclerosis, selectively abolishes one of a co‐chaperone's dual functions. High‐resolution NMR confirmed that the mutation leaves the protein's structure intact while paralyzing its communication with the Hsp70 machinery. The mechanistic results complement *in vivo* studies of ALS disease pathology and present a new strategy to rescue non‐functioning Hsp70 chaperone systems.

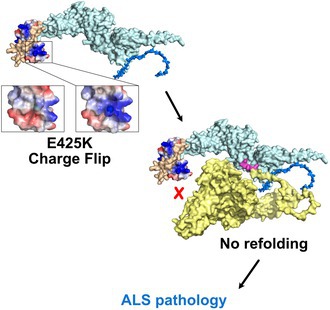

**Abbreviations**
ALS: amyotrophic lateral sclerosis
ATPase: adenine triphosphatase
DNAJC7: DNAJ homolog subfamily C member 7
Hsc70: heat shock cognate 71 kDa
Hsf1: heat shock factor 1
Hsp70: heat shock protein 70 kDa
iPSC: induced pluripotent stem cells
JD: J‐domain
JDP: J‐domain protein
NMR: nuclear magnetic resonance
TDP‐43: TAR DNA‐binding protein 43
TPR: tetratricopeptide repeat
TROSY: transverse relaxation‐optimized spectroscopy

## DNAJC7: A critical relay in the ALS proteostasis network

Amyotrophic lateral sclerosis (ALS) is a fatal neurodegenerative disorder characterized by motor neuron loss and the toxic aggregation of proteins such as TDP‐43. Most cases are sporadic (90–95%) while a handful are familial (5–10%); however, a subset of sporadic cases carries overlapping genetic markers with familial, making the gene origins an important factor for both types of ALS [1]. Among these risk factors, DNAJC7, a Class C J‐domain protein (JDP), has emerged as a vital bridge in the cellular proteostasis network [2].

Structurally, DNAJC7 functions through a bipartite architecture designed for efficient protein quality control: its N‐terminal tetratricopeptide repeats (TPRs) act as “holdases” that grab misfolded clients, while its C‐terminal J‐domain (JD) serves as the “ignition” required to recruit and activate the Hsp70/Hsp90 folding machinery. The importance of this JDP was first highlighted by Farhan *et al*., who identified an enrichment of C‐terminal truncation mutants in ALS patients [3]. These variants lack the J‐domain entirely, providing a clear molecular rationale for the failure of Hsp70‐dependent refolding in these cases.

In the cellular economy, the balance between “energetically expensive” foldases (like Hsp70) and “cheaper” holdases (like DNAJC7) is essential. Holdases play a crucial role by “holding onto” unfolded targets until they can be safely handed off to the refolding machinery, thereby preventing the accumulation of unusable protein during its lifecycle [4, 5]. However, when this relay is disrupted, as seen in DNAJC7 truncations or dysfunctional point mutations, substrates fail to reach the foldase system. This breakdown of the chaperone handoff creates a ripe intracellular environment for the formation of the pathogenic aggregates that define ALS.

## Robust characterization of a single point mutation's impact to structure and function

But the molecular ramifications of the E425K point mutation within the JD remained unclear. Elmaleh *et al*. [6] observed that the global structure of DNAJC7 was maintained in spite of the point mutation, yet the interface of the DNAJC7/Hsc70 was massively disrupted. Binding affinity between the J‐domain of DNAJC7 and Hsc70 shifted from 20 μm for wild‐type to above the upper limit of fluoresence anisotropy (K_d_ >1 mm) with E425K. The authors determined that this negative‐to‐positive charge flip within the JD abolished DNAJC7/Hsc70 binding at the canonical site. However, binding between Hsc70 and DNAJC7 remained, indicating more than one interface present.

By analyzing a series of truncated constructs lacking either the JD or the TPR domains, and utilizing relaxation‐optimized methyl‐TROSY NMR, a technique tailored for high molecular weight systems, the authors mapped a secondary binding interface within the TPR domains that interacts specifically with the C‐terminal EEVD motif of Hsc70. This secondary “anchor” remains fully functional in the E425K mutant. Yet, the study reveals a critical functional bottleneck: Physical proximity via the TPR domains is insufficient for chaperone activity. Without proper interaction with the J‐domain, Hsc70's ATPase engine cannot be ignited, effectively uncoupling the DNAJC7‐Hsc70 folding cycle.

## Consequences for the client: The TDP‐43 “traffic jam”

**Fig. 1:**
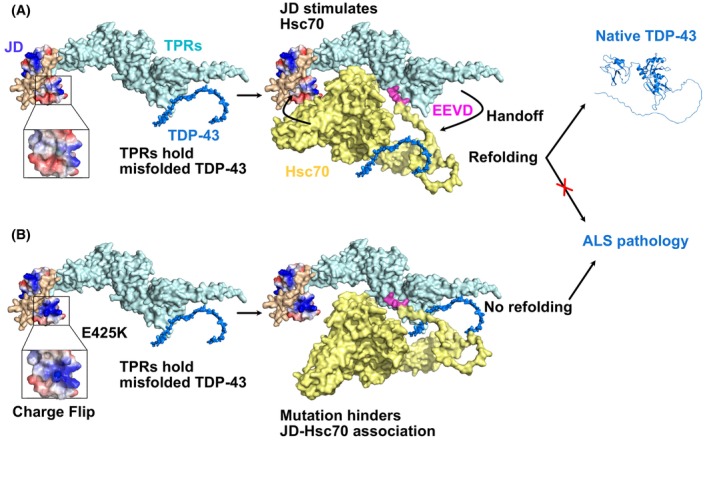
Mechanistic model of the DNAJC7‐Hsc70 relay with TDP‐43 and its disruption in amyotrophic lateral sclerosis (ALS). (A) Wild‐type DNAJC7 captures misfolded TDP‐43 through its tetratricopeptide repeats (TPRs). The correct binding orientation of Hsc70 at the J‐domain (JD) and TPRs enables the handoff of TDP‐43 and the successful refolding process. (B) The E425K mutant retains its ability to hold misfolded TDP‐43, but fails to stimulate the Hsc70. While Hsc70 still anchors to the TPRs by its EEVD tail, the disrupted JD‐Hsc70 interface prevents ATPase stimulation. The lack of refolding activity leads to TDP‐43 aggregation and ALS pathology.

The physiological impact of this uncoupling is most evident when looking at TDP‐43, the hallmark aggregate of ALS. DNAJC7 is supposed to “hold” misfolded TDP‐43 and “hand it off” to Hsc70 for refolding (Fig. 1A). The E425K mutant performs the first half of this task perfectly; it binds TDP‐43 and prevents its immediate phase separation [7]. However, because the J‐domain cannot trigger Hsc70's ATPase engine, the refolding of TDP‐43 never occurs. The authors elegantly demonstrate these impacts using fluorescence‐based phosphate assays, showing the abolishment of ATPase activity with the E425K mutant, and luciferase refolding assays, where E425K DNAJC7 failed to stimulate the refolding of denatured luciferase. In the presence of the E425K mutant, substrates like TDP‐43 remain stuck in a molecular “holding pattern,” unable to access the foldase activity of Hsc70. This results in the protein aggregation and triggers ALS pathology (Fig. 1B).

## A mechanistic study that complements *in vivo* data

Molecular mechanisms remain a driving force of how we understand disease progression, and mechanistic studies bridge the gap between genetic information and the visible impact to cellular pathways. For example, a 2025 study analyzed how DNAJC7 mutations impact the transcriptome and interactome of iPSC motor neurons, showing that the mutated enzyme leads to impaired heat shock factor 1 (Hsf1) activity [8]. However, the study was noted for lacking a mechanistic reason. Hsf1 is known to react not only to heat stress but also to oxidative stress, which is abundant in neurodegenerative disorders such as ALS due to the overgeneration of reactive oxygen species [9]. It is also known that Hsp70 binding to Hsf1 induces trimerization of Hsf1 into its active form as well as stimulates Hsf1/DNA binding activity. Therefore, a plausible missing link between DNAJC7 mutation and loss of Hsf1 activity is the loss of Hsp70 recruitment by the DNAJC7 mutants such as E425K, which in turn contributes to ALS pathology.

Building upon this idea, the loss of ATPase stimulation presents a druggable opportunity within an ALS context; patients possessing the E425K mutant DNAJC7 gene may benefit from therapies which properly activate Hsp70 ATPase activity or replace the protein in the heat shock response (HSR) pathway. The latter idea, conceptually easier to execute, has been investigated in various studies; for example, oxyphenbutazone was recently shown to reduce TDP‐43 aggregation in human neuroblastoma cells without cytotoxicity by upregulating Hsf1 [10], connecting the re‐establishment of HSR pathways—in which Hsp70 participates—to the amelioration of ALS pathology.

## Conclusion

Elmaleh, Faust, and Rosenzweig present a thorough biomolecular study of a single point mutation, linking the shift of canonical to alternative binding sites to downstream protein–protein chaperone nonfunctionality. Their study further exemplifies how solution NMR can elucidate the pivotal role a single mutation plays in an interaction network, providing critical mechanistic insights for a disease model. To capitalize on their information, characterizing the TPR/EEVD interface at atomic resolution within the wild‐type and mutated DNAJC7 could determine which repeats specifically contribute to the interface, providing further insights into this disrupted molecular mechanism.

## Author contributions

T.S.C. and L.G. conceptualized and wrote the original manuscript. T.S.C. designed the graphical abstract. J.B. provided edits.

## Conflict of interest

The authors declare no conflict of interest.
